# Exploring the particle size effect, land use classification, and magnetic characteristics of street dust in urbanized areas in Poland

**DOI:** 10.1038/s41598-025-95972-1

**Published:** 2025-04-14

**Authors:** Sylwia Dytłow, Grzegorz Karasiński

**Affiliations:** Institute of Geophysics Polish Academy of Sciences, Księcia Janusza 64, 01-452 Warsaw, Poland

**Keywords:** Environmental impact, Geomagnetism

## Abstract

Street dust was collected from 149 locations (“all”) and fractionated into five size-dependent categories: (1–0.8 mm) “0.8,” (0.8–0.6 mm) “0.6,” (0.6–0.4 mm) “0.4,” (0.4–0.2 mm) “0.2,” and (< 0.2 mm) “ < 0.2.” The main aims were to investigate the role of grain size in differentiating magnetic properties and identify land use factors affecting the magnetic and grain size distribution of street dust. The mass-specific magnetic susceptibility (χ) for “all” exhibited spatial variability, ranging from 30 to 545 × 10^8^ m^3^/kg. The average χ for “ < 0.2” was 68% higher in Area 1 and 195% higher in Areas 2&3 compared to the “all” samples. The 0.4–0.2 mm grain fraction was the most abundant and comprising 40% ± 7% of the samples in Area 1, 38% ± 9% in Area 2, and 39% ± 12% in Area 3. Area 1 “all” samples predominantly consisted of particles around 1 μm, while samples from Areas 2&3 featured grains ranging between 1 and 5 μm. This study is important as it provides new insights into how grain size and land use factors interact to affect the magnetic properties of street dust, which can be used as an effective indicator for monitoring urban pollution.

## Introduction

Environmental pollution from particulate matter (PM) in highly urbanized areas is a critical issue negatively impacting the global population^1^. According to the United Nations^2^, only 30% of the world’s population lived in urban areas in 1950. However, urbanization has since intensified, contributing to a rise in pollution, particularly from PM deposited on road surfaces. Commonly referred to as “road dust,” “road deposited sediments,” or “street dust,” this material represents a heterogeneous mixture of locally occurring soils and particles originating from both natural and anthropogenic sources.

Road dust pollution poses a significant threat to air quality and public health across various European countries. The origins of road dust vary significantly across different regions and time periods^3^. Geographically, emissions of road dust are particularly high in southern Europe, where the climate tends to be drier, and in Scandinavian countries, where road sanding and the use of studded tires contribute to the road dust pollution^4^. Temporally, road dust emissions are heavily influenced by weather conditions, such as rainfall, sunlight and road moisture^3,5^. In Scandinavian countries i.e. Sweden, Norway, and Denmark, the main sources of traffic-related pollution originate from the abrasion of road surfaces, traction materials, and the wear of vehicle brakes and tires^6^. Amato et al.^3^ identified four main sources of road dust: road wear, motor exhaust, brake wear and tire wear. Road wear was identified as the dominant source in Spain (∼60%), but it constitutes only 30% of road dust in Switzerland, where contributions are more equally distributed among the four main sources of road dust.

The primary source of street dust is the deposition of atmospheric suspended particles, which vary significantly in origin^7^. In Warsaw’s traffic-dominated areas, the dominant source of pollution is traffic-related emissions, driven by a substantial increase in registered vehicles. Over the last decade, the number of cars in Warsaw has risen by 80%, a sharp contrast to many other European cities where vehicle numbers have declined^8,9^. Notably, more than half (56%) of registered vehicles in Poland are between 11 and 20 years old, with an additional 12.6% exceeding 20 years of age^10^. The average age of vehicles on Warsaw’s roads is approximately 13.6 years. According to a report commissioned by the Warsaw city authorities^11^, cars older than 15 years with a diesel engine in 2020 were responsible for 40% of PM pollution in Warsaw.

In areas with lower traffic intensity, the composition of road dust is influenced by pollution sources such as industrial emissions, vehicular activity, and low-stack emissions. Traffic-related emissions—both exhaust and nonexhaust—include pollutants from fuel combustion, wear and tear of vehicle components (e.g., tires, brake pads, and body rust), leakage of lubricating and brake oils, road surface abrasion, catalytic converter wear, and road paint degradation^12–16^.

The main factors affecting road dust can be included in four categories: road characteristics, traffic conditions, land use, and meteorology^3^. Ferrimagnetic iron oxides (e.g., magnetite and maghemite) from both natural and anthropogenic sources commonly occur in the urban environment, and therefore, street dust frequently has strong magnetic properties. It was reported by many researchers that anthropogenic activities and pollution sources (fossil fuel combustion, industrial activity, moving cars, and tire abrasion) generate significant amounts of both heavy metals and magnetic minerals^17–20^. Numerous studies have demonstrated significant correlations between magnetic parameters and various types of environmental pollution, including heavy metals, across different environmental materials such as soil^21–23^, street dust^24^, sediments^25^, plant leaves^26^, and particulate matter^27^.

Lanzerstorfer^18^ reported that 49 out of 177 papers concerned the concentration of the chemical elements depending on particle grain size. Scientists have paid less attention to the systematic investigation of the magnetic and physical properties of a statistically significant amount of street dust samples separated into grain-size fractions. Lanzerstorfer^18^ reports that the papers that analyze the size of road dust grains and the magnetic properties of the fractions most often show studies on 1 or 2 isolated fractions. To date, there is a limited number of studies in which a statistically significant number of road dust samples would be investigated to analyze grain sizes in several fractions. To fill this gap, our publication presents research results for 149 locations in Warsaw, which were then divided into 5 factions of granulometric fractions. In this way, 745 road dust samples were obtained, and magnetic susceptibility was obtained for this set of samples.

The main hypothesis of this study is that grain size plays a significant role in differentiating the magnetic properties of street dust, and that granulometric separation can influence the effectiveness of street dust as an indicator of environmental pollution. Specifically, the study hypothesizes that the magnetic properties, particularly magnetic susceptibility, vary across different granulometric fractions and are influenced by land use factors and traffic intensity.

This study aimed to investigate the role of grain size in differentiating the magnetic properties of street dust and to explore how granulometric separation influences the effectiveness of street dust as an indicator of environmental pollution. The research also sought to identify land use factors that affect the resulting magnetic and grain size distribution of street dust, based on on-site inspections of sampling locations. A novel aspect of the study is the examination of the spatial distribution of magnetic susceptibility across different granulometric fractions, with the goal of identifying hotspots of magnetic susceptibility. Furthermore, the study aimed to define land use factors that influence the analysis of mineralogical composition and hysteresis parameters based on grain size diameter. This approach offers new insights into how grain size and land use factors interact to affect the magnetic properties of street dust, which has implications for monitoring environmental pollution.

## Study area

**Fig. 1:**
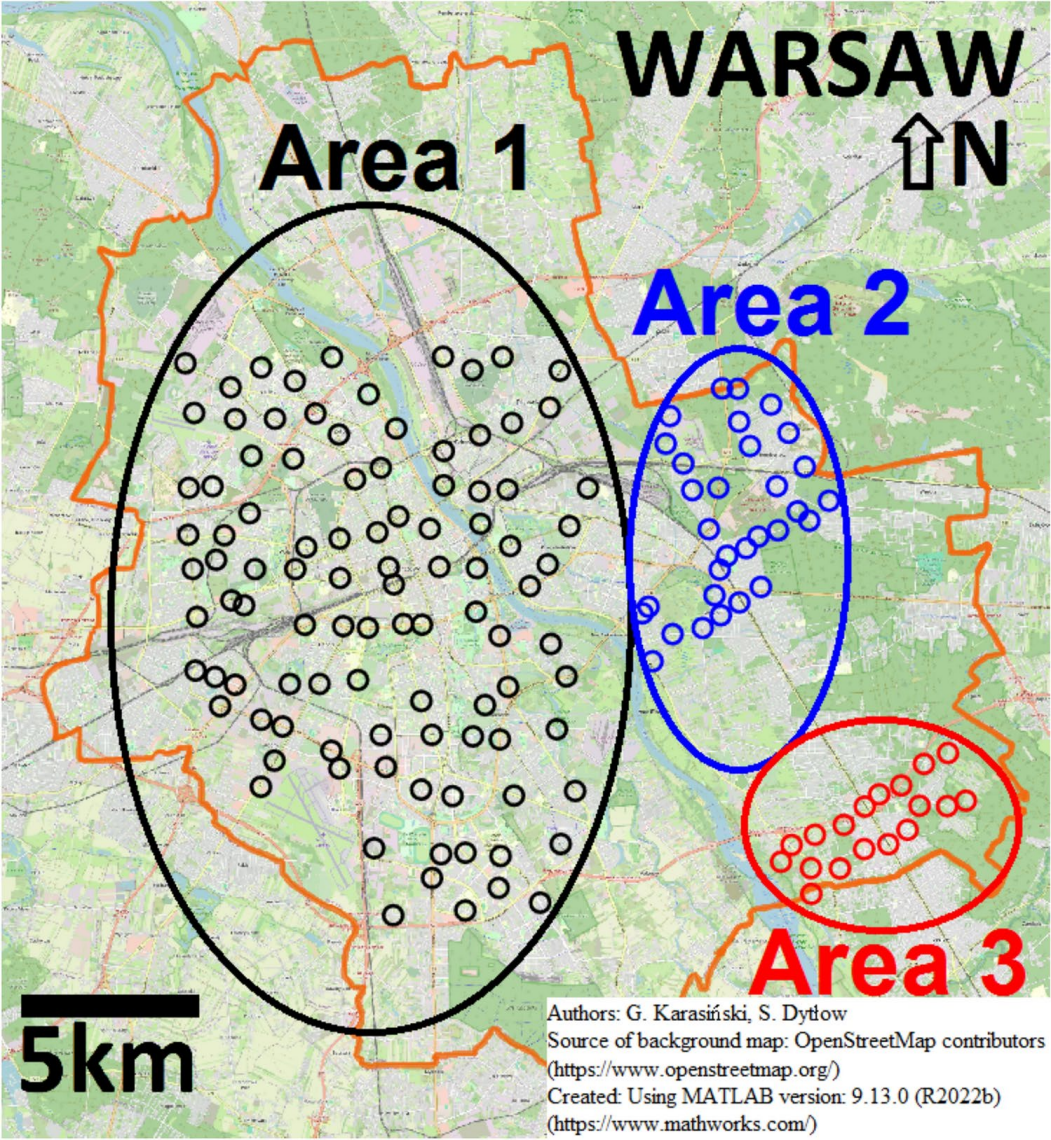
Locations of the 149 sampling sites in Warsaw. Area 1—Central Warsaw districts are marked by black circles, Area 2—Rembertów district is marked by blue circles, and Area 3—Wawer district is marked by red circles. Created using MATLAB version: 9.13.0 (R2022b) (https://www.mathworks.com/); source of background map: OpenStreetMap contributors (https://www.openstreetmap.org/).

The sampling campaigns were conducted in Warsaw, the capital city of Poland, located in the Mazovian region along the Vistula River (Fig. 1). Warsaw spans an area of 517.24 km^2^ and has a population of approximately 1.8 million. Road dust samples were collected from various urban zones within the city, including the central district (Area 1) and peripheral districts—Rembertów (Area 2) and Wawer (Area 3) (Fig. 1).

## Methods

### Sample preparation of street dust

Street dust was collected from 149 sampling sites across the Warsaw urban area in 2023. Sampling locations were geolocated using a handheld global positioning system (GPS). Street dust was sampled using a sanitized plastic vacuum cleaner and by manually sweeping with a brush. Sampling points were selected based on factors such as wind direction and proximity to industrial areas. Each sample was a composite of subsamples collected within a 5-m radius of the sampling point and stored in polyethylene bags.

Sampling was conducted on nonrainy days between September and November 2023. The collected samples were air-dried, sieved, and labeled as “all” for granulometric fractionation. The fractions obtained were then weighed.

The granulometric analysis was carried out using a laboratory shaker model LPzE-2e (MULTISERW-Morek, Poland), equipped with a set of sieves with the following mesh sizes: 1 mm, 0.8 mm, 0.6 mm, 0.4 mm, and 0.2 mm. This allowed for the separation of particle size fractions as follows: particles with a diameter between 1 and 0.8 mm (designated as fraction “0.8”), between 0.8 and 0.6 mm (fraction “0.6”), between 0.6 mm and 0.4 mm (fraction “0.4”), between 0.4 and 0.2 mm (fraction “0.2”), and particles less than 0.2 mm in diameter (fraction “ < 0.2”).

### Description of magnetic methods

#### Magnetic susceptibility measurement (χ)

Magnetic susceptibility (χ) quantifies the ability of materials to alter their magnetization when exposed to an external magnetic field. It depends on the concentration of magnetic particles, their mineralogical composition, and the presence of fine magnetic grains^28,29^. Low-field volume magnetic susceptibility (κ) of each sample was measured at two frequencies—976 Hz and 15,600 Hz—with a sensitivity of 2 × 10^−8^ SI, under a magnetic field strength (H) of 200 A/m. Measurements were conducted using the multifunction Kappabridge MFK1-FA (AGICO, Czech Republic) and normalized by mass. The frequency dependence of χ (χ_fd%_) was calculated as the percentage difference in χ measured at low and high magnetic field frequencies, following the method described by Dearing et al.^30^.

#### Sample preparation for measurements of anhysteretic remanent susceptibility (χ_ARM_) and hysteresis loops

Samples for anhysteretic remanent magnetization (ARM) and hysteresis loop measurements were prepared using cylindrical gelatin capsules (~ 12 mm in length and ~ 6 mm in diameter). Each capsule was filled with a mixture of 450 mg of dust sample and 250 mg of sodium silicate.

Hysteresis loops were measured using a Vibrating Sample Magnetometer (VSM, Molspin, Great Britain) with a maximum applied magnetic field of 1 T. Key magnetic parameters—saturation magnetization (M_S_), saturation remanent magnetization (M_RS_), and coercive field (H_C_)—were determined after correcting for paramagnetic contributions. Additionally, a direct current (DC) back-field of isothermal remanent magnetization (IRM) was measured to calculate the remanent coercivity (H_CR_).

An anhysteretic remanent magnetization (ARM) was acquired using an alternating field (AF) of 100 mT and a direct field (DC) of 100 μT, utilizing the LDA-5 (AGICO) device. ARM measurements were conducted with a JR-6 Dual Speed Spinner Magnetometer (AGICO). The final values of ARM were denoted as the anhysteretic remanent susceptibility (χ_ARM_) by dividing the ARM value by the DC bias magnetic field (*H* = 79.62 A/m).

#### Spatial distribution interpolation method

Two-dimensional (2D) maps of the spatial distribution of χ were generated using the Matlab^31^ function “ScatteredInterpolant” to interpolate values between scattered points. This method employs Delaunay triangulation^32^ and applies the “natural neighbor” interpolation technique^33^ to produce smooth 2D images. The “nearest neighbor” interpolation technique using the Voronoi diagram plane partition method was applied for land use mapping^34^. The resulting 2D images were overlaid in a transparent format onto a Warsaw map derived from the OpenStreetMap basic layer^35^.

#### Thermomagnetic curves of κ(T)

Thermomagnetic curves of κ(T) were measured in an air atmosphere using the Kappabridge KLY-3 coupled with the CS-3 high-temperature furnace, covering a temperature range of 30–700 °C. Curie temperatures (T_C_) were determined using the differential method^36^. Measurements were conducted on both raw samples and magnetic fractions of the dust.

#### Data treatments

Initial data processing, including statistical calculations and table preparation, was performed in Microsoft Excel. Advanced analysis and visualization were carried out using Origin 2019 and Matlab.

## Results and discussion

### Particle mass size distribution

**Fig. 2:**
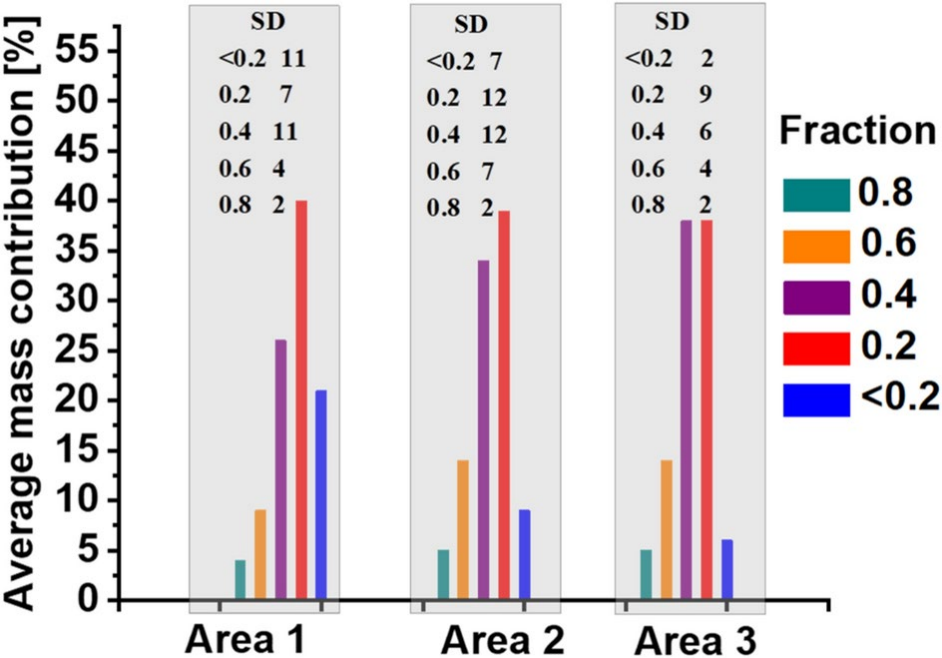
Average mass percentage contributions and standard deviation values (SD) of different granulometric fractions of road dust from Warsaw, as indicated in the legend (fractions: “0.8”, “0.6”, “0.4”, “0.2”, and “ < 0.2 mm”). Histograms represent data for Area 1, Area 2, and Area 3.

The mass percentage distribution of particle size fractions (Fig. 2) reveals that the 0.4–0.2 mm fraction is the most abundant across all investigated areas. In Area 1, the distribution indicates a skew towards finer fractions, with the “0.2” fraction making up 40% ± 7% of the total mass and grains “0.4” contributing 26% ± 11%. Area 2 exhibits a bimodal distribution, with one peak corresponding to “0.4” fraction 34% ± 12% and another to the finer fraction “0.2” 39% ± 12%. In contrast, Area 3**,** reveals a single, shifted peak, with both the “0.2” and “0.4” fractions contributing 38% each. This indicates a more even distribution, without clear bimodality. The largest particle size fraction (1–0.8 mm) represents the smallest contribution to the total sample weight, making up only 4% ± 2% in Area 1, 5% ± 2% in Area 2, and 5% ± 2% in Area 3.

Han et al.^37^ reported that in Baotou, China, the 50–100 µm fraction was the most abundant (59.0%), followed by the 100–300 µm fraction (26.4%). Shi et al.^20^ found a significant unimodal distribution in Shanghai, China, with most particles in the 100–400 µm range. In Turin, Italy, Padoan et al.^38^ noted that the most abundant fraction of street dust was 200–2000 µm (62%), followed by 200–2000 µm grains (27%), whereas the smallest contribution comes from the 2.5–10 µm fraction (2%). Studies from Beijing, China, by Shen et al.^39^ showed a relatively uniform distribution in high-traffic areas, with domination by the 38–74 μm (24% ± 2%) and 125–300 μm (26% ± 2%) fractions. Similarly, Zhao et al.^40^ found that the finest fraction (0–63 μm) contributed only 6.0–13.0% to the total sample mass. For Thessaloniki, Greece, Bourliva et al.^41^ reported a contribution of 8.0–41.3% for the finest fraction (0–63 μm), while Logiewa et al.^14^ found a 14.6–46.8% contribution in Krakow, Katowice, and Olkusz, Poland.

### Spatial distribution of χ of street dust

Mass-specific magnetic susceptibility (χ) of road dust in the study area varies significantly across locations. For “all” samples, χ ranges from 30 to 545 × 10^−8^ m^3^/kg in Area 1 and from 20 to 239 × 10^−8^ m^3^/kg in Areas 2 and 3 (Table S1). The variability in χ values for Area 1 (median: 196 × 10^−8^ m^3^/kg; average value 208 × 10^−8^ m^3^/kg) is broader compared to Areas 2 and 3 (median: 69 × 10^−8^ m^3^/kg; average: 85 × 10^−8^ m^3^/kg) (Table S1). The median χ value in Area 1 is more than twice that of Areas 2 and 3, reflecting the greater variability in land use in Area 1 compared to the predominantly suburban and residential characteristics of Areas 2 and 3^42^.

The average χ (χ_average_) for both Area 1 and Areas 2 and 3 is highest in the “0.8” and “ < 0.2” fractions. Specifically, the χ_average_ for the “0.8” fraction is 36% higher in Area 1 and 229% higher in Areas 2 and 3 compared to the corresponding values for “all” samples (Fig. 4 and Table S1). Similarly, the χ_average_ for the “ < 0.2” fraction is 68% higher in Area 1 and 195% higher in Areas 2 and 3 than the corresponding “all” sample values. These findings suggest that the “0.8” and “ < 0.2” fractions are most enriched with anthropogenic magnetic particles, indicating higher traffic-related pollution levels.

The “ < 0.2” fraction, in particular, may be heavily influenced by the wear of brake pads. Previous studies^43,44^ report that brake emissions are dominated by fine and ultrafine particles (UFPs, < 0.1 μm), which constitute up to 95% of the total particle number. Larger particles in this fraction may result from corrosion processes.

In Area 1, χ values can be categorized into two predominant ranges: 150–200 × 10^−8^ m^3^/kg (29 samples) and 200–250 × 10^−8^ m^3^/kg (26 samples) (Fig. S2). Extreme values, including the minimum and maximum χ intervals (0–50, 400–450, and 500–550 × 10^−8^ m^3^/kg), are represented by only one sample each.

**Fig. 3:**
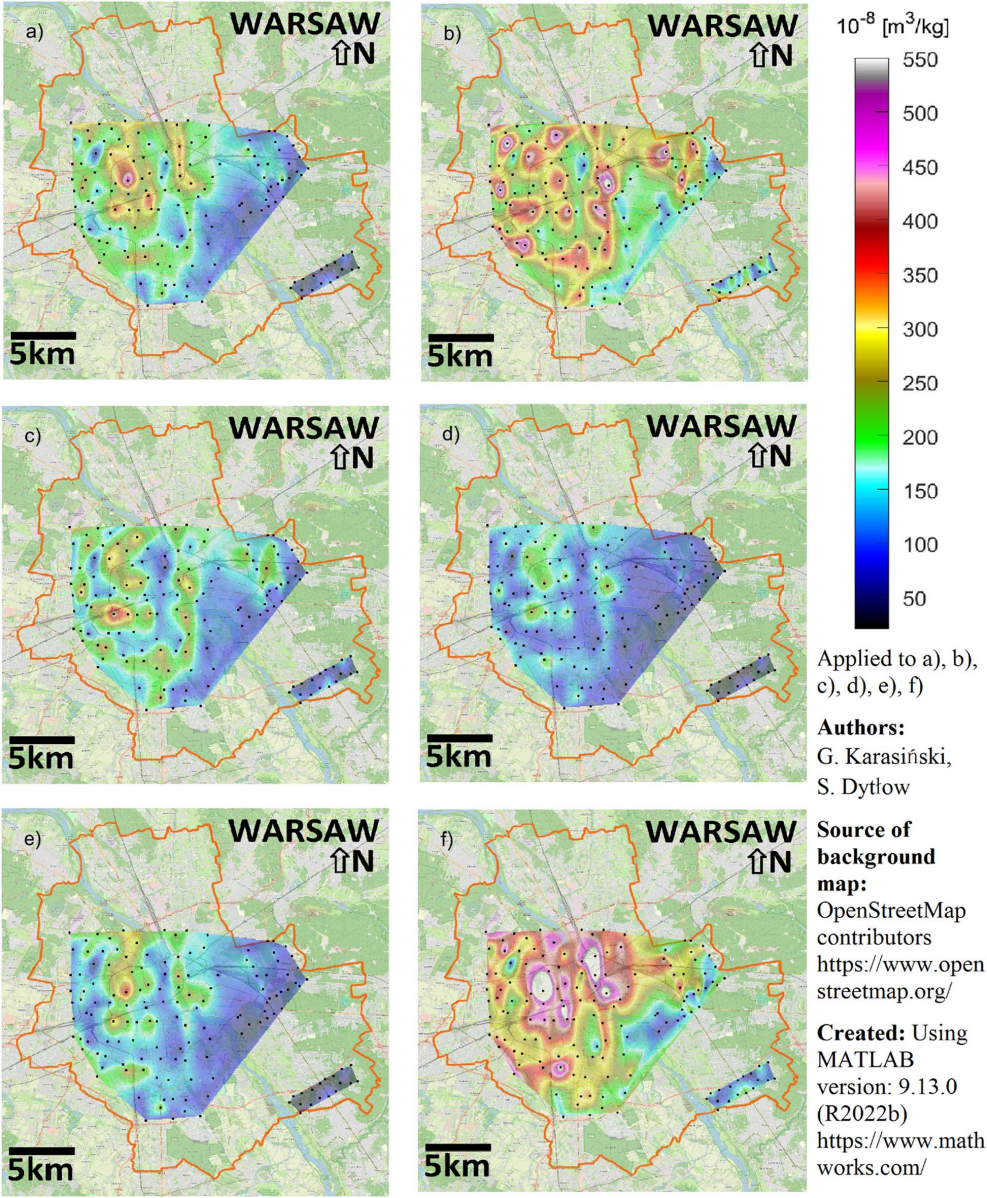
Maps of interpolated spatial χ distribution of street dust over Warsaw (Poland) for different granulometric fractions: (**a**) “all,” (**b**) “0.8,” (**c**) “0.6,” (**d**) “0.4,” (**e**) “0.2,” and (**f**) “ < 0.2”. Created using MATLAB version: 9.13.0 (R2022b) (https://www.mathworks.com/); source of background map: OpenStreetMap contributors (https://www.openstreetmap.org/).

When examining the spatial distribution of extreme χ values, these are predominantly located in high-traffic regions of Area 1, with smaller values observed in Area 3. Figure 3b reflects this pattern across all fractions. However, the “0.8” fraction shows a more uniform distribution, as indicated by the hotspot similarities between Areas 1 and 2 in Fig. 3b. The relative difference in mass χ values between Areas 1 and 2 versus Area 3 is also the smallest for the “0.8” fraction.

**Fig. 4:**
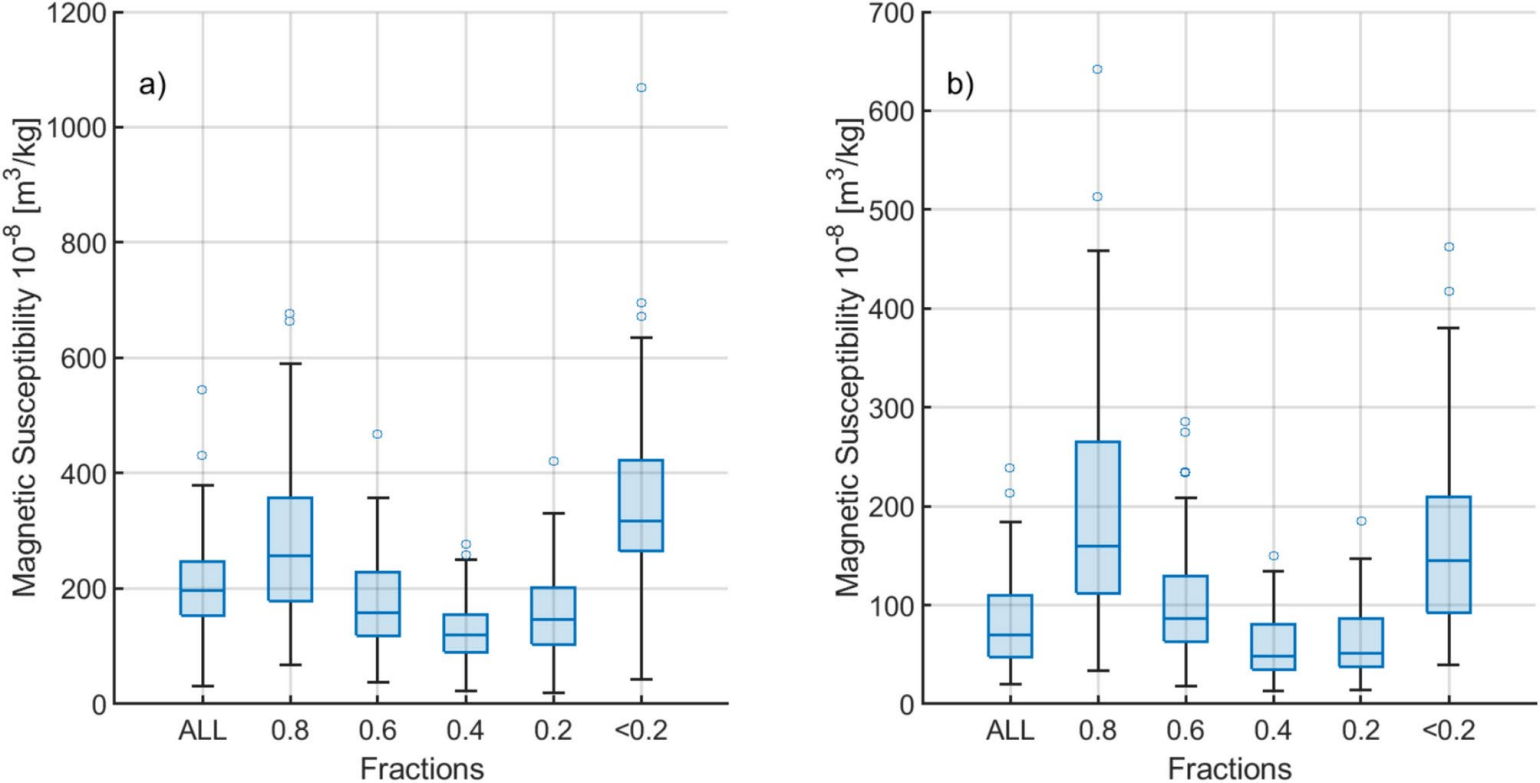
Chartboxes for the χ of granulometric fractions of Area 1 (**a**) and Area 2&3 (**b**).

Figure 4 highlights the concentration of magnetic carriers in the “0.8” and “ < 0.2” fractions, as shown in the box plot comparing χ across fractions and areas. The observation of two maxima—higher χ values in the “ < 0.2” fraction and unexpectedly high values in the “0.8” fraction—suggests at least two distinct sources of magnetic susceptibility carriers.

One source is vehicle emissions, including brake and tire wear, exhaust emissions, and ultrafine particulate matter, which predominantly contribute to the “ < 0.2” fraction. Another source is corrosion from vehicles, infrastructure, and road maintenance machinery, which may account for the coarse particles in the “0.8” fraction. These findings emphasize the diverse origins of magnetic particles in urban street dust.

Figure 3 presents the interpolated spatial distribution of χ for street dust across all studied granulometric fractions: “all,” “0.8,” “0.6,” “0.4,” and “ < 0.2.” Among these, the “0.4” fraction exhibits the lowest χ values and the smallest range between minimum and maximum values. For the “0.4” fraction, χ values range from 22 × 10^−8^ (Area 1) and 13 × 10^−8^ m^3^/kg (Areas 2 and 3) to a maximum of 277 × 10^−8^ m^3^/kg in both areas.

High χ values are primarily associated with traffic-related pollution in central Warsaw (Area 1), particularly along major roads and intersections with heavy traffic. The χ distribution for “all” samples (Fig. 3a) reveals strong maxima in Area 1, ranging from 270 to 350 × 10^−8^ m^3^/kg. The highest χ (545 × 10^−8^ m^3^/kg and 1068 × 10^−8^ m^3^/kg for fraction “ < 0.2”) values were recorded in the Wola district at the Okopowa/Żytnia Street intersection, a dual-lane road with tram tracks and daily traffic of ~ 35,000 vehicles^45^. Generally, for the whole study area, the hotspots of χ are located on the left bank of the Vistula River and attributed to the multilane roads, main crossroads, and sampling sites located near the tram/railway lines. The maximum χ values for “all” (545 × 10^−8^ m^3^/kg) and “ < 0.2” (1068 × 10^−8^ m^3^/kg) fractions were both observed at this hotspot. For the “0.8” fraction, the highest value in Area 1 (676 × 10^−8^ m^3^/kg) was recorded near a petrol station and tram line.

In Areas 2 and 3, the maximum χ (239 × 10^−8^ m^3^/kg) was measured at a roadside sampling point along a medium-traffic street (~ 10,000 vehicles/day) that serves as an exit for heavy-duty vehicles (e.g., a bus terminus). The minimum χ value (20 × 10^−8^ m^3^/kg) in these areas was observed on a gravel road adjacent to a green space. Interestingly, two sampling sites within Area 3 along the same street (870 m apart) showed highly variable χ values for “all” samples (142 × 10^−8^ m^3^/kg vs. 40 × 10^−8^ m^3^/kg), despite similar traffic intensities (< 2000 vehicles/day). This discrepancy suggests that local factors, such as microenvironmental conditions or specific pollution sources, influence χ values^46^.

The results align with findings from Dytłow et al.^22^, who reported χ values for Warsaw street dust collected in 2013 ranging from 49 to 1025 × 10^−8^ m^3^/kg. Comparatively, Wang et al.^47^ found much higher χ values for Shanghai, China—a city ten times larger than Warsaw by area—ranging from 120 to 4040 × 10^−8^ m^3^/kg, with an average and median of 810 and 590 × 10^−8^ m^3^/kg. Other studies in Shanghai^24^ reported χ values from 175 to 3367.3 × 10^−8^ m^3^ kg^−1^, with a mean of 838.7 × 10^−8^ m^3^ kg^−1^.

Similar results were observed in coastal cities of Fujian, China, including Zhangzhou, Xiamen, and Quanzhou, where χ ranged from 21 to 911 × 10^−8^ m^3^ kg^−1^, with an average of 376 × 10^−8^ m^3^ kg^−1^.^46^. Smaller values were noted on Xiamen Island, China^48^, where χ ranged from 25 to 730 × 10^−8^ m^3^ kg^−1^ with a χ_average_ of 250 × 10^−8^ m^3^ kg^−1^.

The χfd% represents the relative abundance of ferrimagnetic grains near the superparamagnetic – single domain (SP/SD) threshold. When χfd% exceeds 4%, the magnetic particles contain a significant proportion of superparamagnetic particles. In contrast, when χfd% is below 4%, the samples contain a low percentage of SP particles^30^. For all sample sets, the average χ_fd_% values are 4% for Area 1 and 5% for Areas 2 and 3, indicating that a significant amount of SP grains is present in the road dust samples. These findings align with previous studies on atmospheric particulates^49^, road-deposited sediments^42^, and street dust^47^.

### Correlation between χ, traffic intensity, and land use categories

Bućko et al.^50^ highlighted traffic volume as a significant factor influencing the variation in χ values between sampling sites. Another potential factor is the geological background and soil type. In Warsaw, the Quaternary surface deposits of the Mazovian region serve as the primary geogenic component of street dust and are predominantly composed of various sand types with differing proportions of loam and clay^51^. However, due to their uniform geological characteristics, this factor is considered less critical for the study area.

In this study, weak correlations were observed between χ and traffic intensity. The weakest correlation (*r* = 0.2) was noted for the “0.8” fraction in Areas 2 and 3. For fractions “ < 0.2” and “all” in Areas 2 and 3, as well as for fractions “all,” “0.8,” and “ < 0.2” in Area 1, the Pearson correlation coefficient was 0.3. These results suggest that while traffic-related pollution is the dominant source, particularly in Area 1, other factors also significantly influence pollutant concentrations in road dust and, consequently, the values of χ.

Each of the 149 samples was classified into one of 19 land use categories, which include six primary types (1–6) and 13 mixed categories (7–19), as shown in Fig. S4 and detailed in Table S6. The classification identifies these 19 categories, with the first six representing the core land use types, while categories 7 to 19 are combinations of two or three of the primary categories. A more detailed description of categories 1–9 is provided in Table S6.

**Fig. 5:**
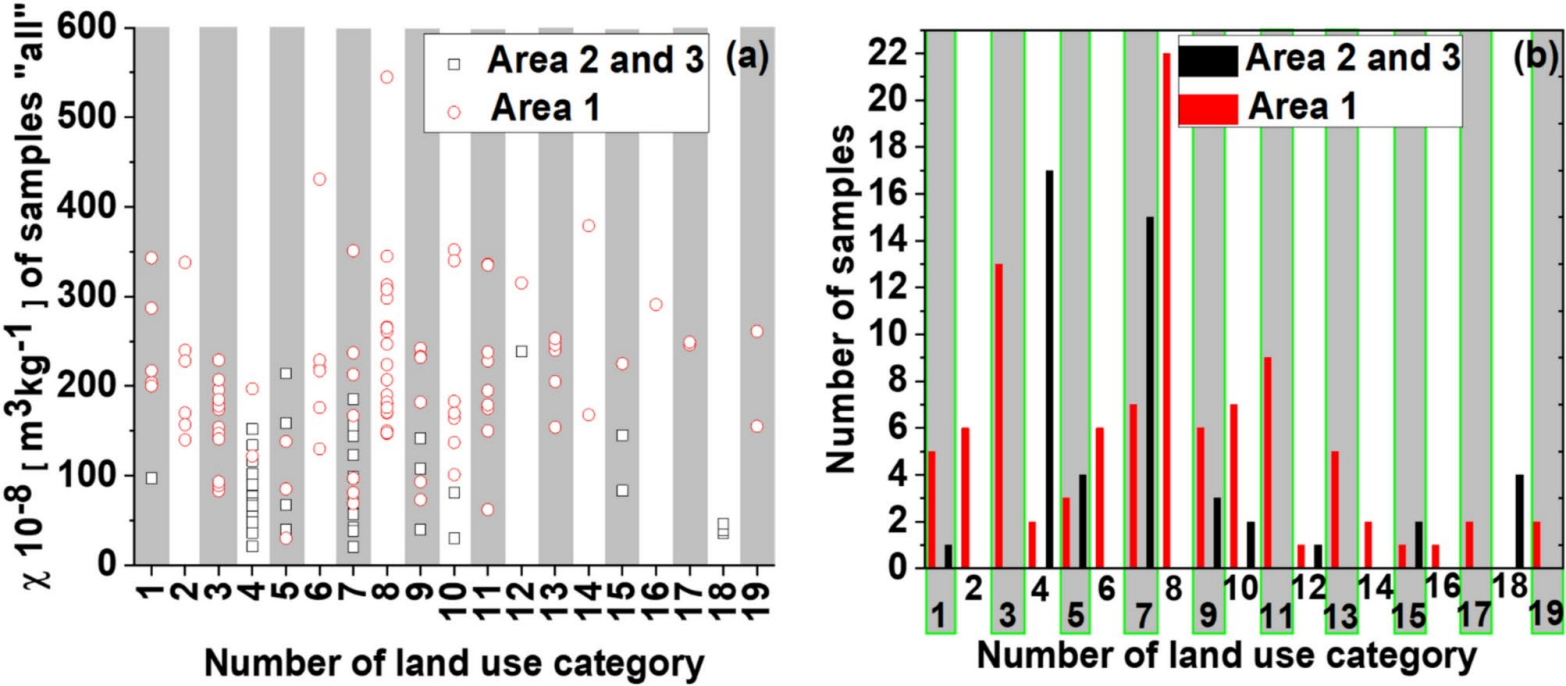
Distribution of samples in 19 land use categories concerning the χ values of “all” samples of street dust from Area 1 (red circles) and Area 2&3 (black squares) (**a**). Count of samples in 19 land use categories (1–19) with a distinction between Area 1 and Area 2&3 (**b**).

In Area 1, 35% of the samples fall into two land use categories: 13% in Category 3 and 22% in mixed Category 8 (Figs. 5b and S3). Areas 2 and 3 display more uniform land use patterns, with 17 out of 49 samples assigned to residential areas (Category 4) and 15 to mixed Category 7 (residential and green areas).

Figure 5a shows χ of the samples belonging to one of the 19 land use categories. The sample with the maximum χ for Area 1 was also classified in category number 8. The next largest land use category for Area 1 is mixed category 11, which accounts for 9% of the samples. This category includes samples with relatively low χ < 250 × 10^−8^ m^3^/kg. A similar pattern of sample classification with the dominance of two land use categories was observed for Areas 2&3. Category 4 and mixed 7 were assigned to 35% (17 samples) and 31% (15 samples), respectively. The maximum χ value for Areas 2&3 (239 × 10^−8^ m^3^/kg) is attributed to mixed Category 12, which is represented by a single sample. This outlier likely reflects unique local conditions, such as a higher density of buildings with varying heights.

Field inspections of the 149 sampling sites reveal significant differences between Areas 1 and 2&3 regarding building heating systems. In Area 1, only 15% of locations lack centralized heating, compared to 94% in Areas 2&3. These findings align with previous χ measurements (Section “Spatial distribution of χ of street dust”), which show higher values in Area 1. For Areas 2&3, this observation is consistent with official data from the Warsaw City Council^52,53^, indicating that nearly all buildings in these areas lack centralized heating (98% in Area 2 and 99.5% in Area 3).

### Parameters of hysteresis loops and remanent coercivity (H_CR_)

**Fig. 6:**
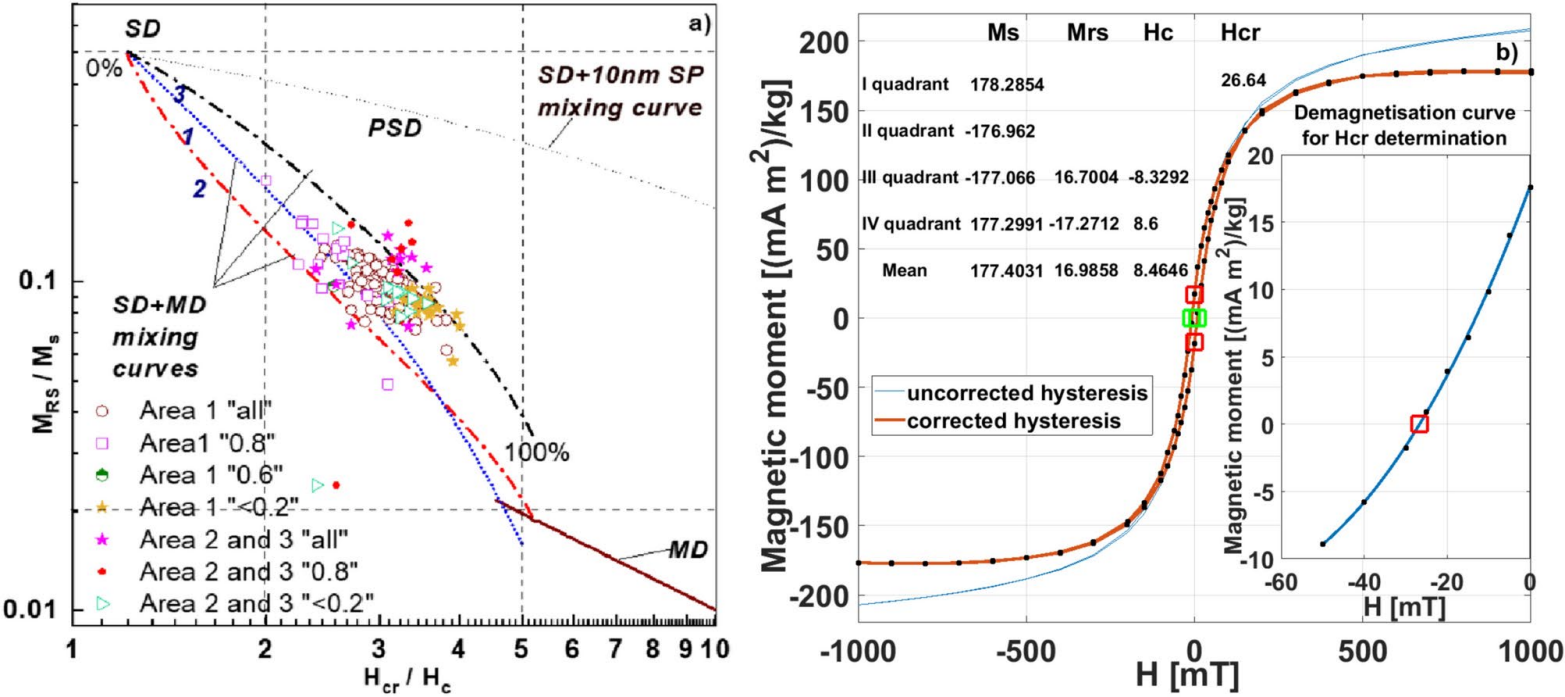
(**a**) The ratios of M_RS_/M_S_ and H_CR_/H_C_ on the Day-Dunlop plot. (**b**) Exemplary hysteresis loop for street dust and back-field demagnetization curve.

Table S5 summarizes the statistical descriptions of the hysteresis loop and H_CR_ parameters. All measured loops were narrow, closed at ~ 100–150 mT, and reached near saturation at ~ 300 mT (Fig. 6b), indicating that ferrimagnetic minerals predominantly contribute to magnetization^22,54–56^.

To further analyze the grain size of magnetic particles, Fig. 6a presents the ratios of hysteresis parameters (Hcr/Hc and Mrs/Ms). The plot of M_RS_/M_S_ vs. H_CR_/H_C_, commonly referred to as the “Day-Dunlop plot”^57–59^ (Fig. 6a), is divided into three regions representing single-domain (SD), pseudo-single-domain (PSD), and multidomain (MD) behavior. Hysteresis data for street dust from all areas and grain size fractions predominantly cluster within the PSD field^57^, aligning with theoretical mixing lines for SD and MD magnetite particles^58,59^.

This finding suggests that the ferrimagnetic minerals in the road dust primarily consist of PSD, which align with previous research on street dust^55^. Notably, coarser granulometric fractions (e.g., “0.8”) tend to plot closer to the SD region, while finer fractions (e.g., “ < 0.2”) align more with the MD field. Additionally, it is observed that some “0.8” and “all” samples from Areas 2 and 3 show slight shifts toward the PSD + SD region and the mixture curve of 10 nm + SP interval.

The back-field demagnetization curves (H_CR_) can be used to determine magnetic composition^60^. For “all” samples, H_CR_ values ranged from 17 to 37 mT (average: 26 mT) in Area 1 and from 10 to 30 mT (average: 26 mT) in Areas 2 and 3 (Table S5). Theoretically, the H_CR_ of SD and MD magnetite is ~ 33 mT and ~ 15 mT, respectively, while that of hematite is ~ 700 mT^29^. The H_CR_ values observed in this study are consistent with those of magnetite, indicating that magnetite is the primary magnetic carrier in the dust samples.

### Correlation analysis between magnetic parameters

**Fig. 7:**
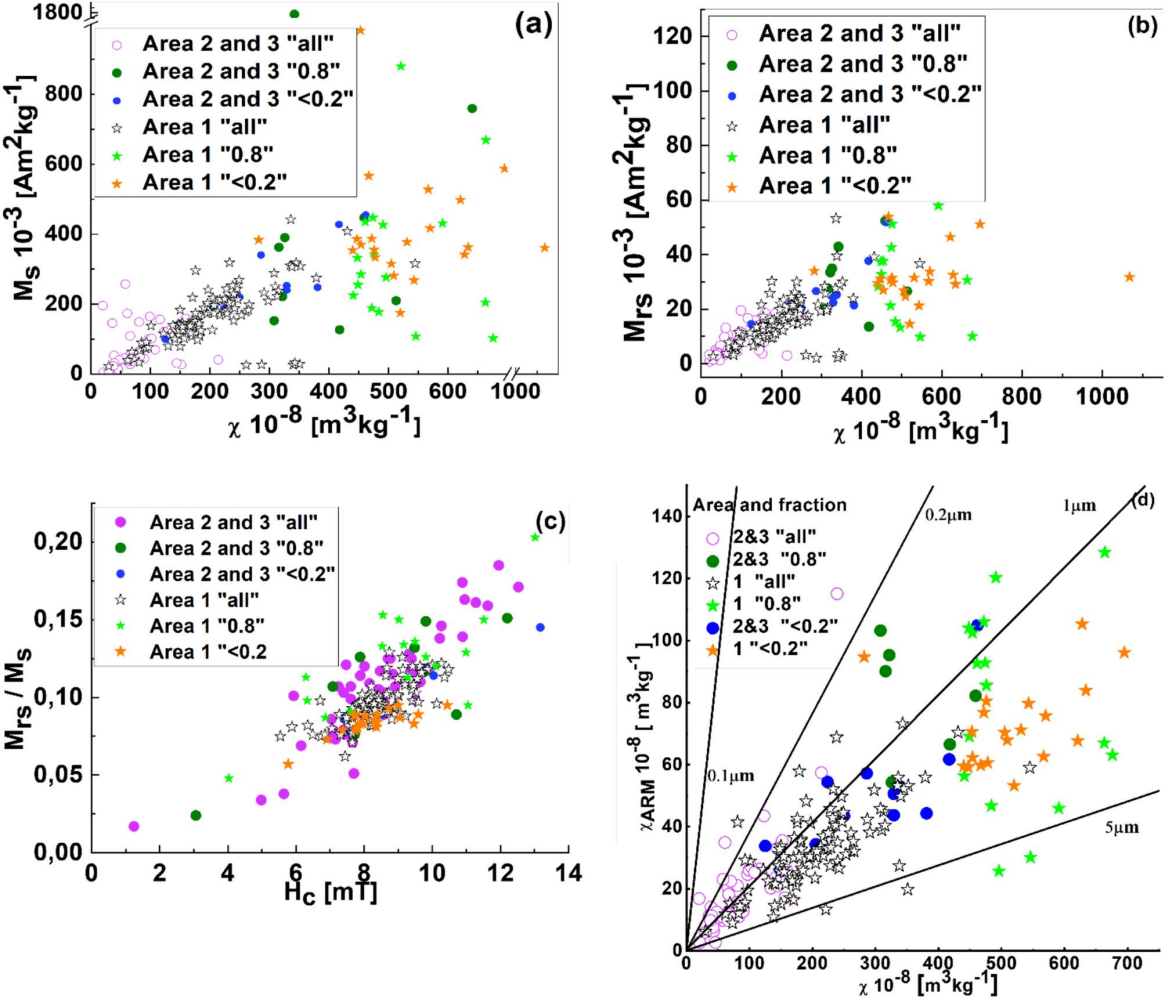
Biplots displaying M_S_ vs. χ (**a**), M_RS_ vs. χ (**b**), M_RS_/M_S_ vs. H_C_ (**c**), χ_ARM_ vs. χ (**d**). Reference lines for plot (**d**) were retrieved from King et al.^62^. The following abbreviations were used on the plots: magnetic susceptibility (χ), anhysteretic remanent susceptibility (χ_ARM_); saturation magnetization (M_RS_); saturation remanence (M_S_); and coercive field (H_C_).

M_RS_ largely reflects the concentration of ferrimagnetic mineral, while χ _ARM_ is particularly sensitive to single domain (SD) magnetic particles^61^. The M_RS_ values ranged from 2 to 53 × 10^−3^ m^2^/kg, with a median of 15 × 10^−3^ m^2^/kg for “all” samples from Area 1 and 5 × 10^−3^ m^2^/kg for Areas 2 and 3 (Table S4). For Area 1, a significant linear correlation between χ and M_RS_ was observed for “all” samples (*r* = 0.62) (Fig. 7b and Table S5), while no correlation was found for the “0.8” and “ < 0.2” fractions. In Areas 2 and 3, strong correlations between χ and M_RS_ were noted for “all” (*r* = 0.62), “0.8” (*r* = 0.73), and “ < 0.2” (*r* = 0.83), indicating that χ variation was primarily controlled by ferrimagnetic particle concentration.

The χ vs. M_S_ plot for “all” samples (Fig. 7a) revealed differences between the areas. Area 1 showed a moderate correlation (*r* = 0.64), while Areas 2 and 3 exhibited a weaker correlation (*r* = 0.38). For the “ < 0.2” fraction, a very strong correlation between M_RS_ and M_S_ was observed, with *r* = 0.96 for Area 1 and *r* = 0.92 for Areas 2 and 3, suggesting heterogeneous mineralogy and/or magnetic grain size.

The strongest correlation between M_RS_/M_S_ and H_C_ (Fig. 7c) was observed for the “ < 0.2” fraction in Areas 2 and 3 (*r* = 0.97), followed by “all” samples (*r* = 0.89). In Area 1, strong correlations were also found for “ < 0.2” (*r* = 0.80) and “all” (*r* = 0.75). As the M_RS_/M_S_ parameter reflects both magnetic mineralogy and grain size, it can be a useful qualitative indicator when combined with H_C_^29^.

The King’s plot (χ_ARM_ vs. χ, Fig. 7d) effectively indicated magnetic grain size^28,62^. For “all” samples, χ_ARM_ varied significantly, ranging from 3 to 115 × 10^−8^ m^3^/kg (average: 83 × 10^−8^ m^3^/kg) in Areas 2 and 3, and from 36 to 73 × 10^−8^ m^3^/kg (average: 33 × 10^−8^ m^3^/kg) in Area 1. Magnetic grain sizes for “all” samples varied between 0.2 and 5 μm. Samples from Area 1 were predominantly aligned with 1 μm grains, while those from Areas 2 and 3 ranged from 1 to 5 μm.

Magnetite-like grains (0.2–5 μm) were attributed to a mixture of coarse SD, fine MD, and PSD grains, as confirmed by the Day-Dunlop plot (Fig. 7a). These PSD and MD grains are commonly associated with industrial activity, fuel combustion, and traffic pollution^15,63^.

### Thermomagnetic curves κ(T)

**Fig. 8:**
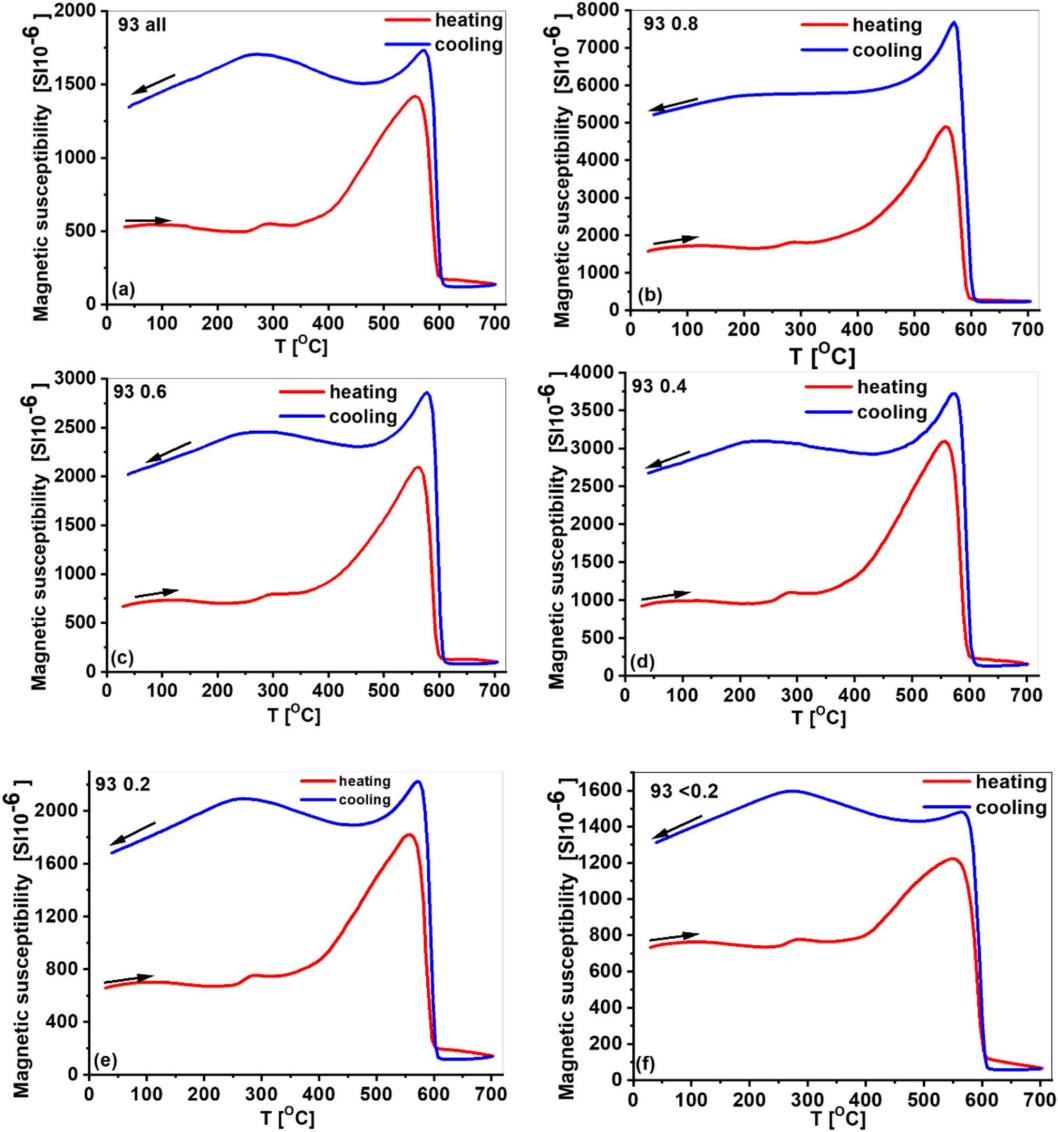
Representative temperature-dependent magnetization (**a**–**f**) curves for a set of 5 granulometric fractions and samples “all” for sample number 93 of street dust. The red and blue lines represent heating and cooling processes, respectively.

Figure 8 shows a set of κ(T) curves measured at representative sample locations, selected based on average χ values. The κ(T) curves for coarse-grained fractions were consistent across locations. The cooling curves were positioned above the heating curves, indicating strong magnetic enhancement following the heating–cooling cycle.

A hump-shaped increase in magnetization was observed on all heating curves around 280–350 °C. This behavior is attributed to the presence of maghemite, which transforms hematite^64^. Alternatively, Van Velzen and Dekkers^65^ suggest that this feature may result from the release of internal stress in low-temperature oxidized magnetite grains.

For magnetic fractions, the magnetization continued to decline beyond ~ 700 °C and did not reach zero, suggesting the presence of metallic iron in the dust with a second Curie temperature above 700 °C (Fig. S6f)^55^. Between 400 and 550 °C, χ increased sharply, with a pronounced peak around ~ 550 °C. This behavior may be due to the reduction of hematite to magnetite in an oxygen-free environment^66^ and/or the Hopkinson effect in magnetite just below its Curie temperature^67^.

On the cooling curves, an increase in κ intensity appeared at T_CMg_, followed by a relatively narrow Hopkinson-like peak and a gradual decline beginning at 400 °C. This could be attributed to the formation of new magnetite from thermally activated chemical alterations of diamagnetic and/or paramagnetic minerals^67^.

## Conclusion

This study examines the role of grain size and land use factors in shaping the magnetic properties and grain size distribution of street dust in urban areas. The results reveal significant spatial variability in magnetic susceptibility (χ) and grain sizes, particularly in different urban areas. The predominant grain size in street dust was in the 0.4–0.2 mm range, with Area 1 (central districts) exhibiting a higher percentage of fine particles (< 0.2 mm) compared to Areas 2 and 3 (residential zones). The magnetic properties, primarily dominated by ferrimagnetic minerals like magnetite, were significantly influenced by land use and traffic intensity. While traffic intensity remains a key factor in shaping magnetic properties, the weak correlation between magnetic susceptibility and traffic volume indicates that other environmental and land-use factors play a substantial role. The spatial distribution of susceptibility highlights specific pollution hotspots, particularly at high-traffic intersections and near tram lines, emphasizing the potential of magnetic methods in identifying localized contamination sources. This study demonstrates the utility of magnetic methods for monitoring urban pollution, offering a practical and cost-effective approach for identifying pollution hotspots. The obtained results could be valuable in determining hotspots using magnetic measurements of street dust, as well as further precision geochemical studies. The relevance of magnetic methods, particularly magnetic susceptibility as a preliminary control tool, is highlighted, offering a promising avenue for environmental monitoring. Overall, this research underscores the importance of magnetic methods in assessing urban pollution dynamics and highlights the potential of street dust analysis as a tool for monitoring and classifying environmental pollution. Moving forward, these findings can inform targeted interventions and policies aimed at mitigating urban pollution and improving environmental quality in cities.

## Supplementary Information

Supplementary Information.

## Data Availability

All data generated or analysed during this study are included in this published article (and its Supplementary Information files).
