# Supplementary material for: Exploring the particle size effect, land use classification, and magnetic characteristics of street dust in urbanized areas in Poland
Source: Sci Rep. 2025 Apr 14;15:12844. doi: 10.1038/s41598-025-95972-1 (PMC11997186; doi:10.1038/s41598-025-95972-1)
Supplement: Supplementary file 1 — Supplementary Information. [file 41598_2025_95972_MOESM1_ESM.docx]

**Exploring the particle size effect, land use classification and the magnetic characteristic of street dust in highly urbanized areas in Europe.**

Sylwia Dytłow^1*^, Grzegorz Karasiński^1^

^1^Institute of Geophysics Polish Academy of Sciences, Ks. Janusza 64, 01-452 Warsaw, Poland

*Corresponding author details: Sylwia Dytłow skdytlow@igf.edu.pl

|  | **AREA 1** | | | | | | |
| --- | --- | --- | --- | --- | --- | --- | --- |
|  | χ 10^-8^m^3^/kg of the street dust fraction | | | | | | χ fd % all |
| Number of samples | 100 | 100 | 100 | 100 | 100 | 100 | 100 |
| Statistical Parameter | all | 0.8 | 0.6 | 0.4 | 0.2 | <0.2 | all |
| Minimum | 30 | 68 | 37 | 22 | 20 | 43 | 0 |
| Maximum | 545 | 676 | 467 | 277 | 422 | 1068 | 6 |
| Average | 208 | 284 | 180 | 127 | 158 | 351 | 4 |
| Median | 196 | 256 | 158 | 119 | 147 | 318 | 4 |
| Third quartile Q_3_ | 246 | 357 | 228 | 154 | 200 | 422 | 4 |
| Standard deviation | 85 | 135 | 83 | 55 | 71 | 147 | 1 |
|  | **AREA 2 and AREA 3** | | | | | | |
|  | χ 10^-8^m^3^/kg of the street dust fraction | | | | | | χ fd % all |
| Number of samples | 49 | 49 | 49 | 49 | 49 | 49 | 49 |
| Statistical Parameter | all | 0.8 | 0.6 | 0.4 | 0.2 | <0.2 | all |
| Minimum | 20 | 34 | 18 | 13 | 14 | 40 | 0 |
| Maximum | 239 | 641 | 286 | 150 | 185 | 462 | 5 |
| Average | 85 | 195 | 105 | 59 | 65 | 166 | 3 |
| Median | 69 | 159 | 86 | 48 | 52 | 145 | 3 |
| Third quartile Q3 | 108 | 262 | 127 | 80 | 86 | 205 | 0 |
| Standard deviation | 50 | 128 | 62 | 32 | 38 | 102 | 1 |

Table S1.  Descriptive statistics of χ of road dust sampled from the study area.


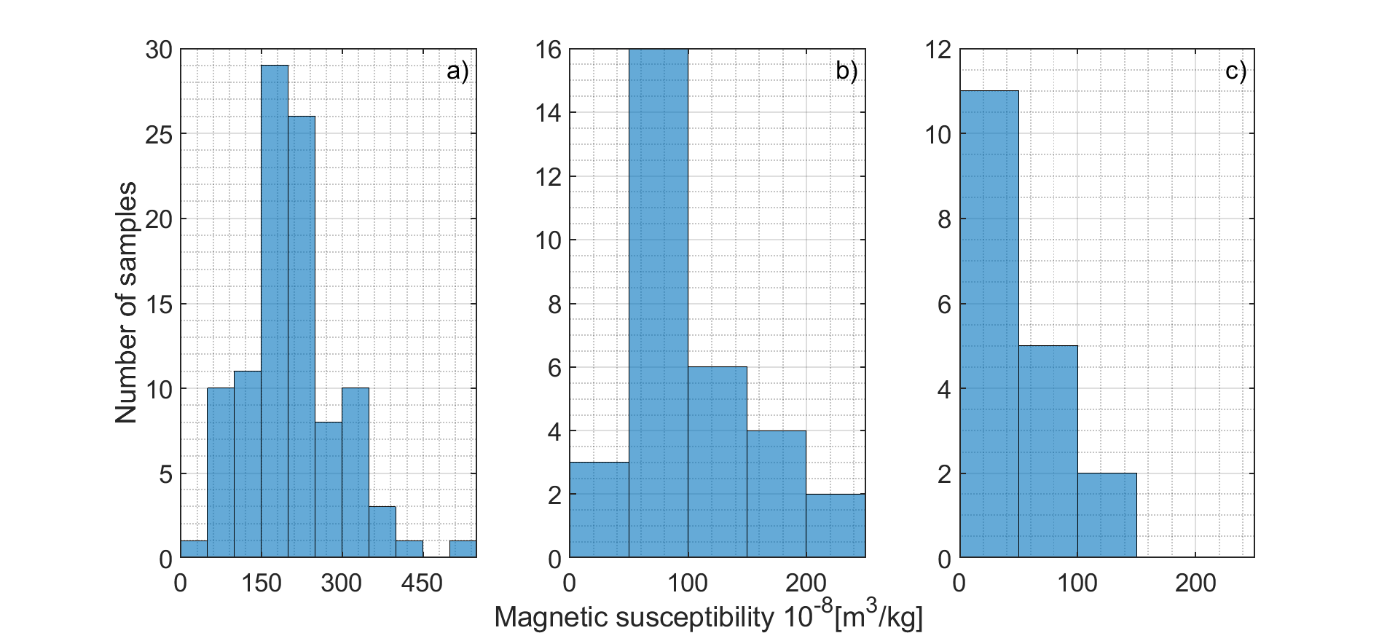


Figure S2. Distribution of the number of samples in the magnetic susceptibility intervals for Area 1 (a), Area 2 (Wawer) (b), Area 3 (Rembertów).


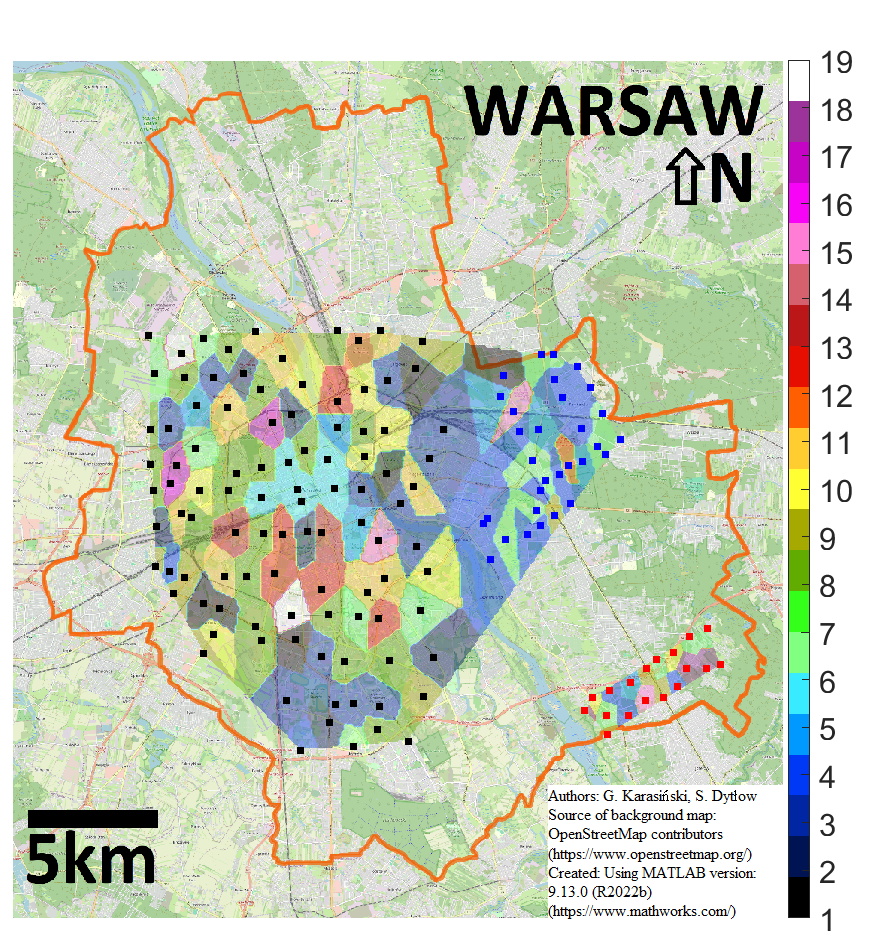


Figure S3. Spatial distribution of 19 land use categories for the 149 sampling sites in Warsaw. Area 1 - Central Warsaw districts are marked by black dots, Area 2 - Rembertów district is marked by blue dots, and Area 3- Wawer district is marked by red dots. Created using MATLAB version: 9.13.0 (R2022b) (<https://www.mathworks.com/>); source of background map: OpenStreetMap contributors (<https://www.openstreetmap.org/>).

|  | **AREA 1** | | | | | | | | | | | | | | |
| --- | --- | --- | --- | --- | --- | --- | --- | --- | --- | --- | --- | --- | --- | --- | --- |
|  | χ_ARM_ 10-8 m3/kg | | | M_RS_ 10^-3^Am^2^/kg | | | M_S_ 10^-3^Am^2^/kg | | | H_CR_ mT | | | H_C_ mT | | |
| Number of samples | 100 | 17 | 21 | 100 | 17 | 21 | 100 | 17 | 21 | 100 | 17 | 21 | 100 | 17 | 21 |
| Statistical Parameter | all | 0.8 | <0.2 | all | 0.8 | <0.2 | all | 0.8 | <0.2 | all | 0.8 | <0.2 | all | 0.8 | <0.2 |
| Minimum | 6 | 26 | 53 | 2 | 10 | 15 | 22 | 102 | 175 | 17 | 12 | 23 | 6 | 4 | 6 |
| Maximum | 73 | 305 | 151 | 53 | 106 | 82 | 442 | 880 | 983 | 37 | 29 | 37 | 10 | 13 | 10 |
| Average | 33 | 91 | 77 | 16 | 43 | 34 | 168 | 340 | 411 | 26 | 22 | 29 | 8 | 9 | 8 |
| Median | 32 | 86 | 71 | 15 | 37 | 31 | 168 | 285 | 370 | 26 | 22 | 29 | 8 | 9 | 8 |
| Third quartile Q_3_ | 41 | 104 | 80 | 21 | 58 | 34 | 215 | 431 | 417 | 27 | 26 | 30 | 9 | 10 | 9 |
| Standard deviation | 14 | 63 | 22 | 9 | 29 | 14 | 84 | 200 | 163 | 3 | 5 | 3 | 1 | 2 | 1 |
|  | **AREA 2 and AREA 3** | | | | | | | | | | | | | | |
|  | χ_ARM_ 10^-8^m^3^/kg | | | M_RS_ 10^-3^Am^2^/kg | | | M_S_ 10^-3^Am^2^/kg | | | H_CR_ mT | | | H_C_ mT | | |
| Number of samples | 49 | 9 | 10 | 49 | 9 | 12 | 49 | 9 | 12 | 49 | 9 | 12 | 49 | 9 | 12 |
| Statistical Parameter | all | 0.8 | <0.2 | all | 0.8 | <0.2 | all | 0.8 | <0.2 | all | 0.8 | <0.2 | all | 0.8 | <0.2 |
| Minimum | 3 | 54 | 26 | 1 | 14 | 14 | 5 | 127 | 100 | 10 | 7 | 7 | 1 | 3 | 7 |
| Maximum | 115 | 522 | 105 | 19 | 113 | 52 | 257 | 1772 | 455 | 30 | 30 | 13 | 13 | 12 | 13 |
| Average | 20 | 152 | 50 | 7 | 40 | 25 | 78 | 493 | 260 | 23 | 22 | 9 | 8 | 9 | 9 |
| Median | 16 | 95 | 47 | 5 | 34 | 22 | 49 | 362 | 244 | 23 | 24 | 9 | 8 | 9 | 9 |
| Third quartile Q3 | 25 | 178 | 54 | 10 | 43 | 26 | 123 | 447 | 320 | 25 | 27 | 9 | 10 | 10 | 9 |
| Standard deviation | 17 | 146 | 22 | 5 | 30 | 138 | 60 | 517 | 1378 | 4 | 7 | 47 | 2 | 3 | 47 |

Table S4. Summary of statistical description of magnetic parameters.

|  | χ "all" Area 1 | χ_ARM_ "all" Area 1 | Ms "all" Area 1 | Mrs "all" Area 1 | Hcr "all" Area 1 | Hc "all" Area 1 | Mrs/Ms "all" Area 1 |  |  | χ "all" Area 2 and 3 | χ_ARM_ "all" Area 2 and 3 | Ms "all" Area 2 and 3 | Mrs "all" Area 2 and 3 | Hcr "all" Area 2 and 3 | Hc "all" Area 2 and 3 | Mrs/Ms "all" Area 2 and 3 |
| --- | --- | --- | --- | --- | --- | --- | --- | --- | --- | --- | --- | --- | --- | --- | --- | --- |
| χ "all" Area 1 | 1.00 |  |  |  |  |  |  |  | χ "all" Area 2 and 3 | 1.00 |  |  |  |  |  |  |
| χ_ARM_ "all" Area 1 | 0.72 | 1.00 |  |  |  |  |  |  | χARM "all" Area 2 and 3 | 0.77 | 1.00 |  |  |  |  |  |
| Ms "all" Area 1 | 0.64 | 0.62 | 1.00 |  |  |  |  |  | Ms "all" Area 2 and 3 | 0.38 | 0.45 | 1.00 |  |  |  |  |
| Mrs "all" Area 1 | 0.62 | 0.59 | 0.96 | 1.00 |  |  |  |  | Mrs "all" Area 2 and 3 | 0.62 | 0.54 | 0.76 | 1.00 |  |  |  |
| Hcr "all" Area 1 | 0.32 | 0.18 | 0.23 | 0.24 | 1.00 |  |  |  | Hcr "all" Area 2 and 3 | 0.13 | -0.07 | -0.16 | 0.04 | 1.00 |  |  |
| Hc "all" Area 1 | 0.16 | 0.12 | 0.17 | 0.32 | 0.71 | 1.00 |  |  | Hc "all" Area 2 and 3 | 0.03 | -0.13 | -0.46 | 0.01 | 0.58 | 1.00 |  |
| Mrs/Ms "all" Area 1 | 0.05 | 0.08 | 0.05 | 0.29 | 0.20 | 0.75 | 1.00 |  | Mrs/Ms "all" Area 2 and 3 | -0.06 | -0.13 | -0.58 | -0.09 | 0.24 | 0.89 | 1.00 |
|  |  |  |  |  |  |  |  |  |  |  |  |  |  |  |  |  |
|  | χ "0.8" Area 1 | χ_ARM_ "0.8" Area 1 | Ms "0.8" Area 1 | Mrs "0.8" Area 1 | Hcr "0.8" Area 1 | Hc "0.8" Area 1 | Mrs/Ms "0.8" Area 1 |  |  | χ "0.8" Area 2 and 3 | χ_ARM_ "0.8" Area 2 and 3 | Ms "0.8" Area 2 and 3 | Mrs "0.8" Area 2 and 3 | Hcr "0.8" Area 2 and 3 | Hc "0.8" Area 2 and 3 | Mrs/Ms "all" Area 2 and 3 |
| χ "0.8" Area 1 | 1.00 |  |  |  |  |  |  |  | χ "0.8" Area 2 and 3 | 1.00 |  |  |  |  |  |  |
| χ_ARM_ "0.8" Area 1 | -0.03 | 1.00 |  |  |  |  |  |  | χ_ARM_ "0.8" Area 2 and 3 | 0.03 | 1.00 |  |  |  |  |  |
| Ms "0.8" Area 1 | 0.05 | 0.82 | 1.00 |  |  |  |  |  | Ms "0.8" Areas 2 and 3 | 0.04 | 0.93 | 1.00 |  |  |  |  |
| Mrs "0.8" Area 1 | 0.07 | 0.76 | 0.92 | 1.00 |  |  |  |  | Mrs "0.8" Areas 2 and 3 | 0.73 | 0.19 | 0.38 | 1.00 |  |  |  |
| Hcr "0.8" Area 1 | -0.24 | 0.34 | 0.41 | 0.50 | 1.00 |  |  |  | Hcr "0.8" Area 2 and 3 | -0.16 | -0.81 | -0.70 | -0.01 | 1.00 |  |  |
| Hc "0.8" Area 1 | -0.19 | 0.37 | 0.44 | 0.62 | 0.90 | 1.00 |  |  | Hc "0.8" Area 2 and 3 | 0.05 | -0.78 | -0.68 | 0.15 | 0.91 | 1.00 |  |
| Mrs/Ms "0.8" Area 1 | 0.02 | 0.28 | 0.28 | 0.62 | 0.49 | 0.75 | 1.00 |  | Mrs/Ms "all" Area 2 and 3 | 0.43 | -0.64 | -0.69 | 0.25 | 0.60 | 0.80 | 1.00 |
|  |  |  |  |  |  |  |  |  |  |  |  |  |  |  |  |  |
|  | χ "<0.2" Area 1 | χ_ARM_ "<0.2" Area 1 | Ms "<0.2" Area 1 | Mrs "<0.2" Area 1 | Hcr "<0.2" Area 1 | Hc "<0.2" Area 1 | Mrs/Ms "<0.2" Area 1 |  |  | χ "<0.2" Area 2 and 3 | χ_ARM_ "<0.2" Area 2 and 3 | Ms "<0.2" Area 2 and 3 | Mrs "<0.2" Area 2 and 3 | Hcr "<0.2" Area 2 and 3 | Hc "<0.2" Area 2 and 3 | Mrs/Ms "<0.2" Area 2 and 3 |
| χ "<0.2" Area 1 | 1.00 |  |  |  |  |  |  |  | χ "<0.2" Area 2 and 3 | 1.00 |  |  |  |  |  |  |
| χ_ARM_ "<0.2" Area 1 | 0.73 | 1.00 |  |  |  |  |  |  | χ_ARM_ "<0.2" Area 2 and 3 | 0.77 | 1.00 |  |  |  |  |  |
| Ms "<0.2" Area 1 | -0.04 | -0.02 | 1.00 |  |  |  |  |  | Ms "<0.2" Area 2 and 3 | 0.89 | 0.84 | 1.00 |  |  |  |  |
| Mrs "<0.2" Area 1 | -0.02 | 0.04 | 0.96 | 1.00 |  |  |  |  | Mrs "<0.2" Area 2 and 3 | 0.83 | 0.96 | 0.92 | 1.00 |  |  |  |
| Hcr "<0.2" Area 1 | -0.39 | -0.07 | 0.40 | 0.56 | 1.00 |  |  |  | Hcr "<0.2" Area 2 and 3 | -0.66 | -0.35 | -0.68 | -0.42 | 1.00 |  |  |
| Hc "<0.2" Area 1 | -0.17 | 0.09 | 0.38 | 0.60 | 0.86 | 1.00 |  |  | Hcr "<0.2" Area 2 and 3 | -0.46 | -0.01 | -0.40 | -0.08 | 0.90 | 1.00 |  |
| Mrs/Ms "<0.2" Area 1 | 0.08 | 0.27 | -0.05 | 0.23 | 0.56 | 0.80 | 1.00 |  | Mrs/Ms "<0.2" Area 2 and 3 | -0.38 | 0.05 | -0.39 | -0.05 | 0.85 | 0.97 | 1.00 |

Table S5. Pearson’s correlation coefficients matrix for magnetic parameters for Area 1 and  Area 2&3. Values were calculated with a significance level alpha=0,05.

| **Number of land-use category** | **Category** | **Deatled description** |
| --- | --- | --- |
| 1 | Industry and Commercial | Areas for manufacturing, factories, warehouses, retail, offices, and service businesses. |
| 2 | Traffic Zones | Infrastructure for roads, railways, public transport, and airports facilitating urban mobility. |
| 3 | High Buildings | Areas with tall buildings (8+ stories), often for offices, mixed-use, or high-density residential. |
| 4 | Low Buildings | Low-rise buildings (1-4 stories), mostly residential or small-scale commercial. |
| 5 | Green Areas | Parks, gardens, open spaces for recreation, environmental conservation, and biodiversity. |
| 6 | City Centre | Urban core with business, cultural, government institutions, and historical sites. |
|  |  | Mixture of primary land use categories |
| 7 | Mixed land use category | 4\5 |
| 8 | Mixed land use category | 2\3 |
| 9 | Mixed land use category | 2\4 |
| 10 | Mixed land use category | 2\5 |
| 11 | Mixed land use category | 3\5 |
| 12 | Mixed land use category | 3\4\5 |
| 13 | Mixed land use category | 2\3\5 |
| 14 | Mixed land use category | 1\2\5 |
| 15 | Mixed land use category | 1\5 |
| 16 | Mixed land use category | 3\4 |
| 17 | Mixed land use category | 1\2 |
| 18 | Mixed land use category | 5\4 |
| 19 | Mixed land use category | 1\3 |

Table S6 Table summarizing the land use categories in Warsaw, including six primary categories (1–6) and thirteen mixed categories (7–19). The mixed categories are combinations of two or three of the primary land use types.
